# Bacterial cell differentiation enables population level survival strategies

**DOI:** 10.1128/mbio.00758-24

**Published:** 2024-05-21

**Authors:** Trisha N. Chong, Lucy Shapiro

**Affiliations:** 1 Department of Pathology, Stanford University School of Medicine, Stanford, California, USA; 2 Department of Developmental Biology, Stanford University School of Medicine, Stanford, California, USA; Instituto Carlos Chagas, Curitiba, Brazil

**Keywords:** cell differentiation, bet-hedging, division of labor, *Caulobacter crescentus*, *Bacillus subtilis*, *Myxococcus xanthus*, signal transduction

## Abstract

Clonal reproduction of unicellular organisms ensures the stable inheritance of genetic information. However, this means of reproduction lacks an intrinsic basis for genetic variation, other than spontaneous mutation and horizontal gene transfer. To make up for this lack of genetic variation, many unicellular organisms undergo the process of cell differentiation to achieve phenotypic heterogeneity within isogenic populations. Cell differentiation is either an inducible or obligate program. Induced cell differentiation can occur as a response to a stimulus, such as starvation or host cell invasion, or it can be a stochastic process. In contrast, obligate cell differentiation is hardwired into the organism’s life cycle. Whether induced or obligate, bacterial cell differentiation requires the activation of a signal transduction pathway that initiates a global change in gene expression and ultimately results in a morphological change. While cell differentiation is considered a hallmark in the development of multicellular organisms, many unicellular bacteria utilize this process to implement survival strategies. In this review, we describe well-characterized cell differentiation programs to highlight three main survival strategies used by bacteria capable of differentiation: (i) environmental adaptation, (ii) division of labor, and (iii) bet-hedging.

## INTRODUCTION

Cell differentiation is a biological process used by multiple bacterial species to implement individual and population-level survival strategies. In this review, we discuss how the process of cell differentiation allows bacteria to implement (i) environmental adaptation, (ii) division of labor, and (iii) bet-hedging strategies.

- Many bacteria respond to environmental changes by differentiating into new cell types that are better suited to their new conditions. Environmental adaptation is implemented at both the individual level through cell-autonomous signaling pathways and at the population level through cell-cell communication (1, 2).
- Division of labor is a population-level survival strategy in which different cell types within a population perform distinct tasks that benefit the population as a whole. This strategy requires individual cells within the population to differentiate into specialized cell types, capable of performing specific tasks. While specialization often comes at a cost to individual cells, this price is outweighed by the increased fitness experienced by the population as a whole (3).
- Bet-hedging is a population-level survival strategy that entails the coexistence of multiple cell types, even some maladapted to the current environmental condition. Bet-hedging lowers the population-level risk by spreading it across multiple different cell types. This strategy is particularly useful to bacteria that experience sudden, unpredictable environmental changes. In this instance, a fraction of the population that was maladapted to the previous condition might be well suited for survival under a new, unanticipated condition (4).

The process of cell differentiation can either be induced or obligate. Induced cell differentiation occurs either stochastically or in response to a stimulus, while obligate cell differentiation is hardwired into the bacteria’s life cycle. *Bacillus subtilis* and *Myxococcus xanthus* undergo induced cell differentiation from dividing cells to metabolically inactive spores upon sensing nutrient depletion (1, 2), while uropathogenic *Escherichia coli* differentiate into coccoid and filamentous morphologies upon host cell invasion (5). *B. subtilis* has been observed to stochastically switch from singlet motile cells to non-motile chaining cells under nutrient-rich conditions (6). In contrast, the aquatic bacterium, *Caulobacter crescentus,* undergoes obligate cell differentiation from a motile swarmer cell to a stationary stalked cell with each cell cycle. While induced cell differentiation is aptly suited for environmental adaptation, *Caulobacter* has been shown to respond to environmental cues by modulating the timing of certain cell cycle events (7–10). Bacteria that undergo either induced or obligate cell differentiation can utilize division of labor and/or bet-hedging strategies. This review will mostly focus on cell differentiation programs in relationship to survival strategies. Further details on each differentiation program can be found in the reviews cited throughout each section.

## INDUCED CELL DIFFERENTIATION FACILITATES ENVIRONMENTAL ADAPTATION

**Fig 1:**
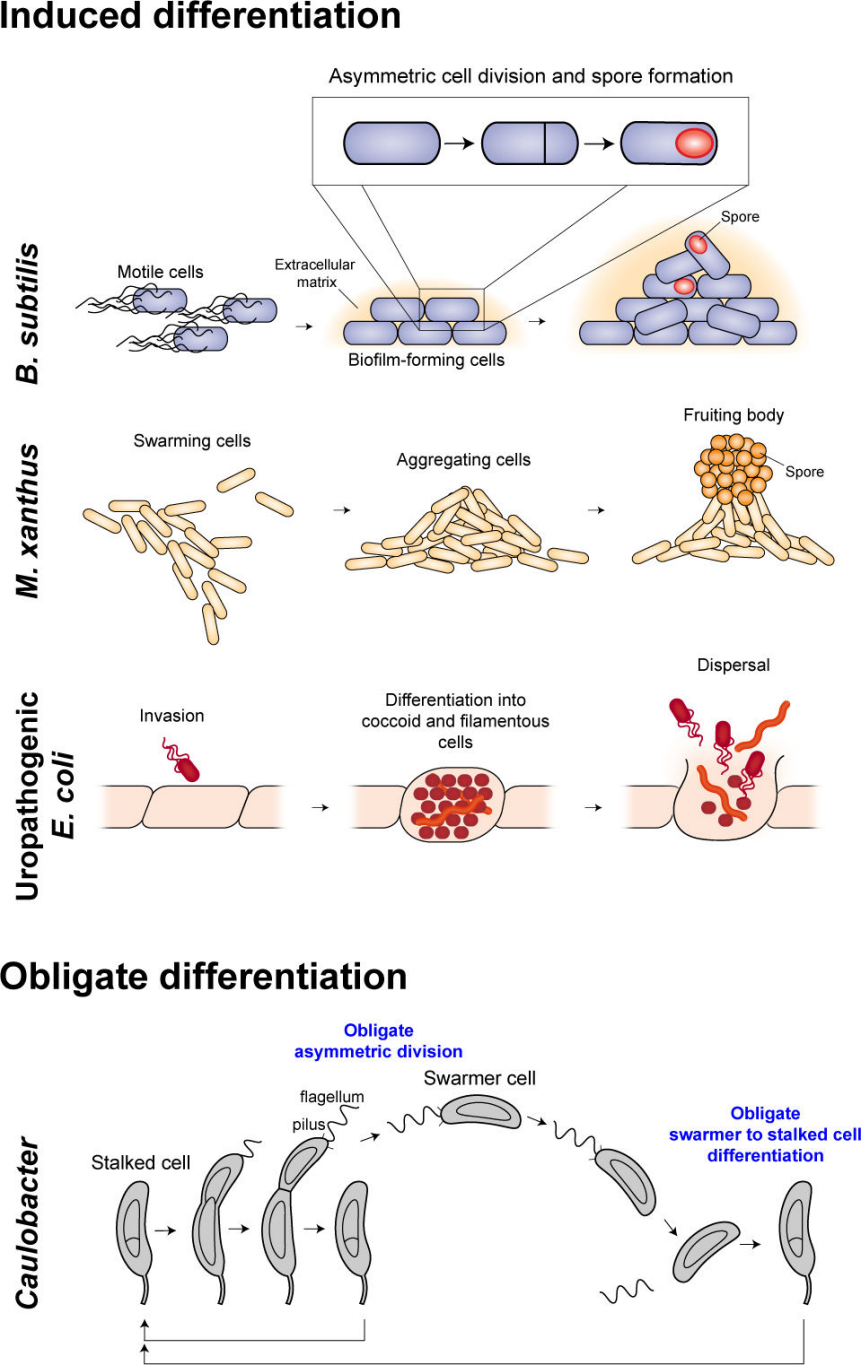
Examples of induced and obligate bacterial cell differentiation. Many bacteria undergo either induced or obligate cell differentiation. Induced cell differentiation is initiated either in response to a stimulus or stochastically. Obligate cell differentiation is hardwired into the organism’s life cycle. Under starvation conditions, *B. subtilis* cells differentiate into biofilm-forming cells and metabolically inactive spores. Sporulation requires a coordinated process of asymmetric cell division and spore formation. *M. xanthus* bacteria also form spores upon nutrient depletion. Swarming *M. xanthus* cells differentiate into aggregating cells that form a fruiting body consisting of spores. *M. xanthus* sporulation does not require cell division, but instead entails the complete remodeling of the cytoskeleton and cell wall. Uropathogenic *E. coli* differentiate into coccoid and filamentous morphologies upon host cell invasion. When infected cells rupture, released bacteria are able to invade neighboring cells, resulting in infection persistence. *Caulobacter crescentus* is an aquatic bacterium that undergoes obligate differentiation from a replication-incompetent motile swarmer cell to a replicating stationary stalked cell, with each cell cycle. Asymmetric division and cell differentiation are both obligate events, hardwired into *Caulobacter’s* cell cycle.

Cell differentiation has been independently employed as a strategy to withstand environmental adversity by both the gram-positive bacterium, *B. subtilis,* and the gram-negative bacterium, *M. xanthus*. Both species form metabolically inactive spores that are protected by a thick coat of proteins and polysaccharides, allowing them to withstand harsh environmental conditions such as starvation, drought, and extreme temperatures (Fig. 1) (2, 11, 12). *B. subtilis* spore formation requires sequential asymmetric cell division and cell differentiation. This developmental program is initiated by phosphorylation and activation of the master transcription factor, Spo0A (13). Phosphorylation of Spo0A is driven by five histidine kinases, KinA–KinE, which respond to various stimuli (14, 15). Upon compartmentalization, large and small daughter cells initiate distinct gene expression programs, specific to each cell fate (11, 16). At a defined point in this developmental program, the large daughter cell engulfs the small daughter cell, allowing the small cell to fully develop into a spore within the cytoplasm of the large cell (11, 17).

**Fig 2:**
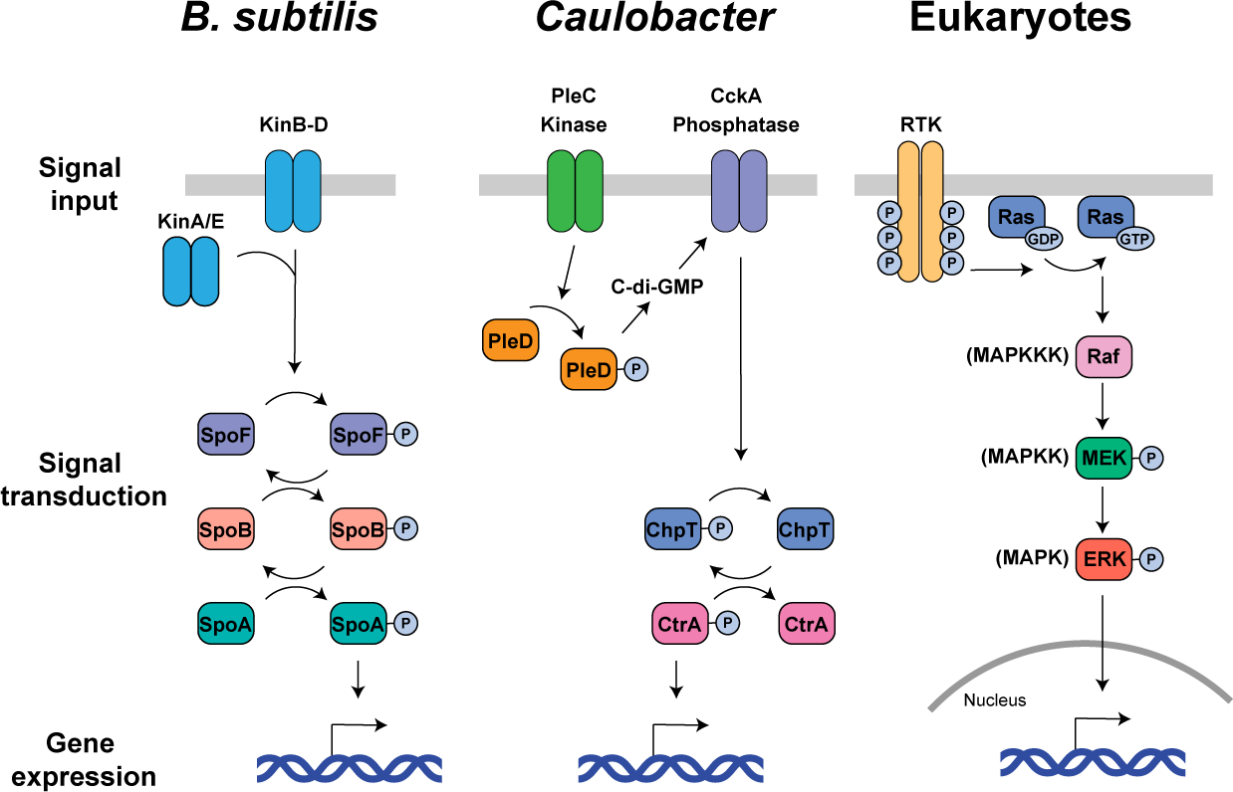
The basic framework of signal transduction pathways is broadly conserved. Signal transduction is required in cells across all kingdoms in order to integrate external environmental and internal physiological signals into biological outcomes such as cell differentiation, cell cycle progression, and cellular adaptation. *B. subtilis* and *Caulobacter* use two-component systems to integrate signals into the decision of whether or not to differentiate. These signaling pathways are built into complex molecular networks that are not depicted in their entirety here. Receptor tyrosine kinase (RTK)/Ras/mitogen-activated protein kinase (MAPK) signaling pathways are an example of eukaryotic signaling pathways that integrate signals into cell fate and cell proliferation decisions. While the specific proteins and their interactions are not conserved between bacterial and eukaryotic signal transduction pathways, the basic framework of these pathways shares many similarities.

Spo0A phosphorylation is responsible for initiation of both biofilm formation and sporulation survival strategies. Biofilm formation is a strategy used by many bacteria to increase tolerance to chemical and physical stressors as well as to exchange metabolites and other common goods between cells. Moderate levels of phosphorylated Spo0A (Spo0A~P) leads to matrix secretion and biofilm formation, while high levels of Spo0A~P is required for sporulation (13, 18). Activation of one or more of the Kin histidine kinases leads to Spo0A phosphorylation either directly or indirectly by a phosphorelay through the response regulator, Spo0F, and the phosphotransfer protein, Spo0B (Fig. 2) (14). KinA and KinB have been previously identified as the main kinases responsible for sporulation, while KinC and KinD have been linked to biofilm formation (14). Analysis of purified proteins revealed that KinA is a more efficient kinase than either KinC or KinD *in vitro* (14, 19). Therefore, activation of specific Kin kinases tunes the level of Spo0A~P to initiate differentiation into either biofilm-forming or spore-forming cell fates.

While the Kin histidine kinases have been extensively studied, little is presently known about the specific stimuli that activate these kinases. Previous studies have suggested that the sporulation-associated kinases, KinA and KinB, likely respond to changes in growth rate or respiration, rather than to external environmental stimuli (20, 21). In contrast, the biofilm-associated kinases, KinC and KinD, likely respond to *B. subtilis*-secreted substances such as matrix components and surfactants (2, 22). These studies suggest that activation of the Kin kinases might be secondary to the molecular pathways responsible for directly sensing changes in environmental conditions. Further investigation into the mechanisms involved in perceiving environmental cues and relaying information to the Kin kinases is required to fully understand how *B. subtilis* differentiation programs are initiated in response to environmental changes. The ability of *B. subtilis* to differentiate into biofilm-forming and spore-forming cells in response to environmental changes ultimately allows the bacteria to survive under extreme conditions.

In contrast to *B. subtilis,* spore formation in *M. xanthus* does not involve cell division. Instead, individual rod-shaped cells completely remodel their cytoskeleton and secrete coat components in order to differentiate into spherical spores (12). Under nutrient-rich, vegetative conditions, cells swarm radially outward, collectively feeding on prey bacteria (1). Upon nutrient depletion, cells undergo a highly coordinated process of aggregation, fruiting body formation, and sporulation that ultimately allows the species to persist through different environmental conditions (Fig. 1) (1, 3, 23). It has been shown that the developmental program responsible for fruiting body formation initiates in reaction to an intracellular accumulation of the stringent response alarmones (p)ppgpp (24, 25). Therefore, similar to *B. subtilis* sporulation*,* the molecular pathway responsible for *M. xanthus* differentiation senses a starvation-induced change in cell physiology rather than nutrient limitation itself. Subsequent to (p)ppgpp synthesis, cells synthesize and release a quorum-sensing A-signal required for cell aggregation (26). Once the A-signal surpasses a threshold level, cells re-direct their motility, aggregating into a protruding mound that develops into a large (~0.08 mm) structure that is the fruiting body (Fig. 1) (27). Within the fruiting body, only ~1%–15% of cells differentiate into spores, while ~80% undergo programmed cell death, and ~5% remain as non-aggregating peripheral rod cells (28, 29). It has been speculated that this large-scale cell death is an example of bacterial altruism (28). Transition from a population of vegetative, predatory cells to a fruiting body consisting of spores requires initiation of an intricate molecular program acting at the individual and population levels. This coordinated program of cell aggregation, fruiting body formation, and sporulation ultimately allows *M. xanthus* to survive through periods of harsh environmental conditions.

Pathogenic bacteria use cell differentiation to facilitate their survival inside infected hosts (5). Upon invasion of bladder epithelial cells, uropathogenic *Escherichia coli* (UPEC) transition from rod to coccoid morphology (5). In addition, a small subset of UPEC cells adopt a long, filamentous morphology that hinders phagocytosis, allowing these cells to evade the host immune system (Fig. 1) (30, 31). Infected epithelial cells eventually rupture, releasing bacteria into the extracellular space. The released bacteria are able to invade neighboring epithelial cells, leading to persistence of the infection (Fig. 1) (5). Many other pathogenic bacteria differentiate into filamentous cell morphologies upon host infection, including *Mycobacterium tuberculosis* and *Salmonella typhimurium* (32, 33). Understanding the signaling pathways associated with these differentiation programs can provide putative targets for decreasing the virulence of these bacterial pathogens.

## PHENOTYPIC HETEROGENEITY ENABLES DIVISION OF LABOR AND BET-HEDGING STRATEGIES

While the process of induced cell differentiation is especially suited for environmental adaptation, species that employ this type of cell differentiation are not precluded from utilizing division of labor or bet-hedging strategies. It has been proposed that *B. subtilis* and *M. xanthus* use both of these population-level survival strategies (3, 4). Division of labor involves distinct cell types performing specialized tasks that benefit the population as a whole. These tasks are divided among individual cells in a way that bears a population-level fitness advantage. Therefore, bacteria are able to bypass the evolutionary pressure put on individual cells by prioritizing the success of the population. In a system in which individual cells expend resources to produce public goods or services, one can imagine that cells that do not act cooperatively can exploit the efforts of the surrounding cells. The emergence of exploitative cells may be less prevalent in monocultures where cells are considered to have a “common goal.” While this review focuses on isogenic populations, cooperation has been documented in multi-species bacterial communities. Therefore, division of labor might be a survival strategy that is employed by both clonal populations and multi-species communities (34, 35).

*B. subtilis* biofilms comprise a consortium of matrix-secreting cells, exoprotease-producing cells, sporulating cells, motile cells, and competent cells, capable of acquiring new DNA (2). Matrix-secreting cells provide a service to the population by producing and secreting matrix components that are required for biofilm integrity. Exoprotease-producing cells secrete proteases that degrade proteins into peptides which can be taken up by cells within the biofilm (36). This type of specialization comes at a cost as specialized cells must expend energy to provide these services. This energetic cost is outweighed by the emergent benefits that cooperation provides to all the cells within the population. In isogenic populations, spores also provide a service as their presence helps to ensure the long-term success of the population. *M. xanthus* spores and non-sporulating cells have been observed to participate in a seemingly one-sided cooperation. In fruiting bodies, aggregating cells form a stalk-like structure that props up the spores. Placement of spores at the end of this cell-made stalk structure has been proposed to aid in spore dispersal (37). Therefore, seemingly altruistic cooperation between various cell types brings forth an increased probability of success for the entire population.

In addition to division of labor, bacteria utilize bet-hedging as another population-level survival strategy. Bet-hedging is a strategy that lowers the population-level risk by spreading it across multiple cell types, even some that are maladapted to the current environmental condition (4). While sporulation is initiated in response to starvation conditions, only a small fraction of *B. subtilis* cells enter the spore-forming cell fate (38, 39). To deter neighboring cells from differentiating, sporulating cells release signaling factors that not only prevent nearby cells from initiating sporulation, but also cause cell lysis. Metabolites from lysed cells are used by their siblings to continue to grow and divide, in a process described as “cannibalism” (40). These dividing cells are poised to initiate rapid growth in case of nutrient influx (41). Conversely, dormant spores are able to persist through prolonged harsh conditions, continuing the genetic legacy of the population. In this way, the population sets itself up for optimal success in the event of any unforeseen changes in nutrient availability.

Bet-hedging is also observed under nutrient-rich conditions. Exponentially growing *B. subtilis* populations consist of singlet motile cells and chains of non-motile cells joined at the poles by extracellular matrix. Motile cells are thought of as foragers, able to seek new nutrient sources, while non-motile chains take full advantage of the current niche (6). Non-motile chains also serve as starting points for biofilm formation, should environmental conditions change (42). Entry into the chaining cell fate is a stochastic process, dictated at its core by just two proteins, the SinR transcriptional repressor and its antagonist, SinI. When free and active, SinR binds to and represses the transcription of genes related to the chaining phenotype. SinR repressor activity is inhibited by direct binding by SinI. Small fluctuations in the relative abundance of SinR and SinI proteins are enough to drive stochastic entry into a SinI dominant state, corresponding with the chaining phenotype (43, 44). Interestingly, the reverse switch from chaining to motile state is not stochastic and relies on a molecular feedback loop that encodes a generational memory, committing cells to the chaining phenotype for a stereotypical number of generations before disassembly into singlet motile cells (45). Constant switching between these two cell types allows *B. subtilis* populations to simultaneously explore and colonize new habitats while being poised to initiate biofilm formation in the event of any unforeseen environmental changes.

How are different gene expression programs initiated in a population of cells that are experiencing relatively the same environment? While heterogeneous micro-environments caused by nutrient and oxygen gradients can explain some level of phenotypic heterogeneity, it has been proposed that many bacteria utilize noise to generate mutually exclusive phenotypic states (46). Positive and negative feedback loops enable small amounts of noise to amplify into large changes in magnitude, resulting in activation or repression of a bistable switch. Such feedback loops within differentiation programs have been proposed to give rise to multiple cell types within isogenic populations (46, 47). As discussed previously, activation of one or many of the KinA–E kinases leads to phosphorylation of the master regulator, Spo0A, responsible for sporulation. In addition, Spo0A~P itself participates in feedback loops that breed further Spo0A synthesis and phosphorylation. For example, Spo0A~P stimulates transcription of both its own gene and that of the upstream response regulator, Spo0F (Fig. 2) (48, 49). While starvation induces Spo0A phosphorylation, it curiously also leads to the synthesis of phosphatases that act on Spo0A~P and Spo0F~P. It has been shown that dephosphorylation of Spo0A~P and Spo0F~P is critical for garnering phenotypic bistability (38, 50, 51). In addition to activating developmental programs associated with biofilm formation and spore formation, Spo0A~P inhibits transcription of the *fla/che* operon, required for the motile cell fate (52). In this way, small amounts of noise in the Spo0A signaling pathway give rise to motile, biofilm-forming, and spore-forming cells, within the same population.

## OBLIGATE DIFFERENTIATION ALLOWS FOR DIVISION OF LABOR AND BET-HEDGING STRATEGIES

Unlike induced cell differentiation, obligate differentiation is hardwired into an organism’s life cycle. The aquatic bacterium, *Caulobacter crescentus,* is studied as a model bacterium that undergoes obligate cell differentiation and asymmetric cell division with each cell cycle (53). Motile, replication-incompetent swarmer cells undergo differentiation into sessile, replicating stalked cells (Fig. 1). This developmental program involves shedding of the polar flagellum, pili retraction, degradation of the chemotaxis machinery, initiation of genome replication, and biogenesis of the stalk appendage (54). These developmental events result from an intricate signaling cascade, initiated by activation of the PleC histidine kinase (Fig. 2) (55–57). PleC phosphorylates the diguanylate cyclase, PleD, allowing for rapid synthesis of the small molecule, cyclic di-GMP (c-di-GMP) (58). This rapid increase in intracellular c-di-GMP concentration leads to dephosphorylation and degradation of the master transcription factor, CtrA (Fig. 2) (59, 60). As phosphorylated CtrA binds to the origin of replication, thereby inhibiting replication initiation, CtrA dephosphorylation and degradation is required to initiate chromosome replication (61). c-di-GMP synthesis additionally triggers the histidine kinase, ShkA, prompting the onset of the stalk biogenesis program (62). Therefore, activation of the PleC kinase initiates an intricate signaling cascade that ultimately results in swarmer to stalked cell differentiation (55, 57).

Conversely, *Caulobacter* asymmetric cell division involves phosphorylation and activation of CtrA in pre-divisional cells (59, 63, 64). Once phosphorylated and active, CtrA promotes the transcription of over 90 developmental genes, many involved in the development of the new flagellar pole (65). Distinct biomolecular condensates positioned at the stalked and flagellar poles allow asymmetric localization of cellular components prior to cell division (66–69). *Caulobacter’s* bi-phasic cell cycle gives rise to two distinct sub-populations: planktonic swarmer cells and stationary stalked and pre-divisional cells. This bi-phasic cell cycle allows for both division of labor and bet-hedging strategies. Division of labor is achieved by stationary cells performing the tasks of proliferation and biofilm formation, while swarmer cells are responsible for dispersal. While this specialization may seem trivial as many other species of bacteria are able to simultaneously disperse and replicate, this division of labor brings forth an emergent advantage for *Caulobacter*. In low-nutrient environments, bacteria must be prudent in their resource allocation. Halting replication allows swarmer cells to persist through various conditions and disperse to new habitats prior to committing to genome replication and matrix secretion, which is required for biofilm formation. In addition, biofilm formation and proliferation exclusively by stalked and pre-divisional cells maintains the integrity of already established communities. These established communities serve as proliferation centers whereby newly born swarmer cells disperse.

Bet-hedging is built into the *Caulobacter* cell cycle as the two cell types are specifically suited to withstand distinct challenges. For example, stalked cells are particularly suited to withstand phage infection as they lack flagella and pili, known sites of phage attachment (70, 71). Additionally, they possess an outer lipopolysaccharide capsule that covers and obscures the proteinaceous S-layer, another phage attachment site (72). In contrast, only swarmer cells are able to enter the persister cell fate, in which cells experience increased antibiotic resistance (73). *Caulobacter’s* continual cycles of colonization and dispersal provide an additional form of bet-hedging. The constant establishment of new communities diminishes the population-level impact associated with the destruction of previously established communities. Utilization of these two strategies, division of labor and bet-hedging, enables *Caulobacter* to thrive in fluctuating, low-nutrient environments such as fresh-water lakes and streams (74). This bi-phasic lifestyle is not only employed by bacteria. Some eukaryotic organisms have convergently evolved life cycles with obligate free-living and sessile phases, including sea jellies and colonial tunicates. Interestingly, these metazoans live in ocean environments where nutrient availability is often low and inconsistent (75, 76).

While obligate cell differentiation is encoded in the *Caulobacter* cell cycle, the process of cell differentiation can be either stimulated or stalled, depending on environmental cues. Multiple studies have reported that the process of swarmer to stalked cell differentiation can be stimulated by surface attachment. In this instance, signal transduction through either the polar pili or flagellum initiates cell differentiation (7, 8, 10). It has also been reported that under starvation conditions, differentiating swarmer cells initiate stalk biogenesis but halt stalk elongation, resulting in a stunted stalk appendage. These cells fail to initiate chromosome replication, not fully committing to the stalked cell fate (9, 77). Therefore, while cell differentiation is obligate to the *Caulobacter* cell cycle, timing of this differentiation event is modulated in response to various environmental cues. In this way, *Caulobacter* is capable of environmental adaption by tuning the duration of specific cell cycle events.

### Conservation among cell differentiation signaling pathways

Both induced and obligate cell differentiation processes enable employment of population-level survival strategies. While activation of these two types of differentiation programs differ, all cell differentiation programs require initiation of a signal transduction pathway. Two-component systems are responsible for most signal transduction in prokaryotes. They consist of a histidine kinase that either phosphorylates or dephosphorylates a response regulator, that then either directly or indirectly enacts a change in gene expression (78, 79). In *B. subtilis,* differentiation into biofilm-forming or spore-forming cells occurs as a result of activation of one or more of the Kin histidine kinases (Fig. 2). Increase in phosphorylation of the master regulator, Spo0A, either directly or indirectly by the Kin kinases, enacts changes in gene expression that result in cell differentiation (14, 15). Obligate swarmer to stalked cell differentiation in *Caulobacter* results from activation of the histidine kinase, PleC, which phosphorylates the response regulator, PleD (55–57). Phosphorylated PleD synthesizes c-di-GMP, indirectly leading to dephosphorylation and degradation of the master regulator, CtrA (Fig. 2) (59, 60). This signaling pathway ultimately results in a change in gene expression, initiation of chromosome replication, and stalk biogenesis.

While two-component systems are found rarely in eukaryotes (although there are reported examples in plants [80]), the basic framework of many eukaryotic signaling pathways shares similarities with those of bacteria. For example, once activated, receptor tyrosine kinase (RTK)/Ras/mitogen-activated protein kinase (MAPK) pathways transmit a signal through a series of protein-protein interactions and post-translational modifications, ultimately resulting in a change in gene expression (81, 82). Typically, when an RTK is bound to a ligand, it undergoes transphosphorylation, in which each dimer subunit phosphorylates tyrosine residues on the other subunit. The resulting multi-phosphorylated protein domain enables docking of signaling proteins which leads to conversion of Ras-GDP to Ras-GTP. Active Ras-GTP stimulates a cascade of MAPK activation and phosphorylation events that results in a change in gene expression (Fig. 2) (83). RTK/Ras/MAPK signaling pathways are used by many eukaryotic cell types to control cell proliferation and cell differentiation (84). While the individual proteins involved in eukaryotic signaling pathways are not homologous to those found in bacterial signaling pathways, the basic framework of many prokaryotic and eukaryotic signaling pathways is quite similar.

## CELL CYCLE-DEPENDENT ACTIVATION OF A SIGNAL PATHWAY INITIATES OBLIGATE DIFFERENTIATION

Signal transduction pathways are designed to initiate in response to a signal. So, how is obligate *Caulobacter* cell differentiation initiated in the absence of an external stimulus? The signal transduction pathway responsible for swarmer to stalked cell differentiation is initiated by PleC kinase activation (55). Therefore, PleC kinase must be activated in a cell cycle-dependent manner to ensure proper timing of swarmer to stalked cell differentiation. PleC kinase has been shown to be activated through three distinct but coordinated cell cycle-dependent events: (i) dissociation and degradation of the PodJ polar complex (57), (ii) subcellular localization of the DivK signaling protein (55), and (iii) retraction of the polar pili (56). When present, the polar scaffold protein, PodJ, localizes to the flagellar pole and inhibits PleC kinase activity (85, 86). Upon PodJ proteolysis and dissociation from the cell pole, PleC is released from the pole and freed from PodJ inhibition (57). At this time, the signaling protein, DivK, is localized to the same pole, where it allosterically binds to PleC, stimulating PleC kinase activity (55). Pili retraction at the same pole increases the PilA concentration in the inner membrane, which binds to PleC and further stimulates PleC activity (56). Therefore, at least three distinct cell cycle-dependent events coordinate the molecular signaling pathway responsible for swarmer to stalked cell differentiation with the rest of the *Caulobacter* cell cycle.

Progression through the *Caulobacter* cell cycle is dictated by seven master regulators, whose peak abundance oscillates out of phase with each other. These master regulators are responsible for initiating specific gene expression programs associated with cell cycle events such as flagellum assembly or chromosome segregation (87). Acting in concert with these core master regulators are spatially resolved signaling circuits that, once initiated, execute cell cycle events at the correct time and location. Each signaling circuit acts as a functional module that communicates with other modules both to indicate their completion and to signal for the initiation of the next module (88). The three signaling events that contribute to swarmer to stalked cell differentiation provide an example of how seemingly independent modules coordinate cell cycle progression. Prior to swarmer to stalked cell differentiation, the PodJ polar complex is dissociated from the cell pole through displacement by another polar scaffold protein, SpmX (66). At this time, PodJ is also degraded by the proteases PerP and MmpA (89). Once PodJ is removed from the cell pole, PleC is freed from kinase inhibition. Stimulation of PleC kinase activity through allosteric binding of the phosphotransfer protein, DivK, requires a high concentration of DivK at the cell pole. While DivK is abundant in swarmer cells, it is not localized to the cell pole until it is recruited by SpmX through interaction with the histidine kinase, DivJ (90, 91). In addition to PleC, DivJ also phosphorylates PleD, further ramping up the intracellular concentration of c-di-GMP (55). Therefore, PleC release from kinase inhibition and kinase stimulation depends on PodJ removal and SpmX localization at the incipient stalked pole. Lastly, the PleC kinase is further stimulated by direct binding of PilA to PleC’s N-terminal transmembrane domain upon retraction of the polar pili. This additional stimulation coordinates PleC kinase activity with the deconstruction of a swarmer cell-specific polar appendage (56). Therefore, multiple signaling pathways feed into PleC kinase activation to initiate swarmer to stalked cell differentiation. Input from these three signaling pathways allows for modulation at multiple levels and coordination of swarmer to stalked cell differentiation with the rest of the cell cycle.

## CONCLUSIONS

Many species of bacteria have evolved cell differentiation programs. Whether to survive the onset of harsh environmental conditions, to adapt to life inside a host, or to employ division of labor or bet-hedging strategies, cell differentiation has been implemented by a multitude of bacterial species to excel in various niches. Both induced and obligate cell differentiation programs have come about by organizing seemingly simple two-component systems into intricate molecular networks. Cell differentiation in eukaryotes is achieved through signal transduction pathways that are divergent from those in bacteria but accomplish the same goal of coordinating signal input with differential gene expression and morphological change. In this review, we described well-characterized examples of bacterial cell differentiation. Beyond these examples, there is undoubtedly a wide array of bacterial differentiation programs that result in various cell fates, contributing to the widespread success of bacteria over billions of years.

## References

[1] Kaiser D, Robinson M, Kroos L. 2010. Myxobacteria, polarity, and multicellular morphogenesis. Cold Spring Harb Perspect Biol 2:a000380. doi:10.1101/cshperspect.a000380 20610548 PMC2908774

[2] Lopez D, Vlamakis H, Kolter R. 2009. Generation of multiple cell types in Bacillus subtilis . FEMS Microbiol Rev 33:152–163. doi:10.1111/j.1574-6976.2008.00148.x 19054118

[3] van Gestel J, Vlamakis H, Kolter R. 2015. Division of labor in biofilms: the ecology of cell differentiation. Microbiol Spectr 3:MB–0002. doi:10.1128/microbiolspec.MB-0002-2014 26104716

[4] Morawska LP, Hernandez-Valdes JA, Kuipers OP. 2022. Diversity of bet-hedging strategies in microbial communities—recent cases and insights. WIREs Mech Dis 14:e1544. doi:10.1002/wsbm.1544 35266649 PMC9286555

[5] Justice SS, Harrison A, Becknell B, Mason KM. 2014. Bacterial differentiation, development, and disease: mechanisms for survival. FEMS Microbiol Lett 360:1–8. doi:10.1111/1574-6968.12602 25228010 PMC4227932

[6] Losick R, Desplan C. 2008. Stochasticity and cell fate. Science 320:65–68. doi:10.1126/science.1147888 18388284 PMC2605794

[7] Ellison CK, Kan J, Dillard RS, Kysela DT, Ducret A, Berne C, Hampton CM, Ke Z, Wright ER, Biais N, Dalia AB, Brun YV. 2017. Obstruction of pilus retraction stimulates bacterial surface sensing. Science 358:535–538. doi:10.1126/science.aan5706 29074778 PMC5805138

[8] Hershey DM, Fiebig A, Crosson S. 2021. Flagellar perturbations activate adhesion through two distinct pathways in Caulobacter crescentus. mBio 12:1–17. doi:10.1128/mBio.03266-20 PMC7885107 33563824

[9] Britos L, Abeliuk E, Taverner T, Lipton M, McAdams H, Shapiro L. 2011. Regulatory response to carbon starvation in Caulobacter crescentus. PLoS One 6:e18179. doi:10.1371/journal.pone.0018179 21494595 PMC3073932

[10] Hug I, Deshpande S, Sprecher KS, Pfohl T, Jenal U. 2017. Second messenger–mediated tactile response by a bacterial rotary motor. Science 358:531–534. doi:10.1126/science.aan5353 29074777

[11] Piggot PJ, Hilbert DW. 2004. Sporulation of Bacillus subtilis. Curr Opin Microbiol 7:579–586. doi:10.1016/j.mib.2004.10.001 15556029

[12] Müller FD, Schink CW, Hoiczyk E, Cserti E, Higgs PI. 2012. Spore formation in Myxococcus xanthus is tied to cytoskeleton functions and polysaccharide spore coat deposition. Mol Microbiol 83:486–505. doi:10.1111/j.1365-2958.2011.07944.x 22188356 PMC3832110

[13] Strauch M, Webbt V, Spiegelmant G, Hoch JA. 1990. The SpoOA protein of Bacillus subtilis is a repressor of the abrB gene. Proc Natl Acad Sci U S A 87:1801–1805. doi:10.1073/pnas.87.5.1801 2106683 PMC53571

[14] LeDeaux JR, Yu N, Grossman AD. 1995. Different roles for kinA, KinB, and KinC in the initiation of sporulation in Bacillus subtilis. J Bacteriol 177:861–863. doi:10.1128/jb.177.3.861-863.1995 7836330 PMC176674

[15] Jiang M, Shao W, Perego M, Hoch JA. 2000. Multiple histidine kinases regulate entry into stationary phase and sporulation in Bacillus subtilis. Mol Microbiol 38:535–542. doi:10.1046/j.1365-2958.2000.02148.x 11069677

[16] Chareyre S, Li X, Anjuwon-Foster BR, Updegrove TB, Clifford S, Brogan AP, Su Y, Zhang L, Chen J, Shroff H, Ramamurthi KS. 2024. Cell division machinery drives cell-specific gene activation during differentiation in Bacillus subtilis. Proc Natl Acad Sci U S A 121:e2400584121. doi:10.1073/pnas.2400584121 38502707 PMC10990147

[17] Crawshaw AD, Serrano M, Stanley WA, Henriques AO, Salgado PS. 2014. A mother cell-to-forespore channel: current understanding and future challenges. FEMS Microbiol Lett 358:129–136. doi:10.1111/1574-6968.12554 25105965

[18] Branda SS, González-Pastor JE, Ben-Yehuda S, Losick R, Kolter R. 2001. Fruiting body formation by Bacillus subtilis. Proc Natl Acad Sci U S A 98:11621–11626. doi:10.1073/pnas.191384198 11572999 PMC58779

[19] Jiang M, Tzeng YL, Feher VA, Perego M, Hoch JA. 1999. Alanine mutants of the Spo0F response regulator modifying specificity for sensor kinases in sporulation initiation. Mol Microbiol 33:389–395. doi:10.1046/j.1365-2958.1999.01481.x 10411754

[20] Kolodkin-Gal I, Elsholz AKW, Muth C, Girguis PR, Kolter R, Losick R. 2013. Respiration control of multicellularity in Bacillus subtilis by a complex of the cytochrome chain with a membrane-embedded histidine kinase. Genes Dev 27:887–899. doi:10.1101/gad.215244.113 23599347 PMC3650226

[21] Kiehler B, Haggett L, Fujita M. 2017. The PAS domains of the major sporulation kinase in Bacillus subtilis play a role in tetramer formation that is essential for the autokinase activity. Microbiologyopen 6:1–12. doi:10.1002/mbo3.481 PMC5552956 28449380

[22] Aguilar C, Vlamakis H, Guzman A, Losick R, Kolter R. 2010. Kind is a checkpoint protein linking spore formation to extracellular-matrix production in Bacillus subtilis Biofilms. mBio 1:e00035-10. doi:10.1128/mBio.00035-10 20689749 PMC2912670

[23] Murphy P, Comstock J, Khan T, Zhang J, Welch R, Igoshin OA. 2023. Cell behaviors underlying Myxococcus xanthus aggregate dispersal . mSystems 8:mSystems doi:10.1128/msystems.00425-23 PMC10654071 37747885

[24] Manoil C, Kaiser D. 1980. Accumulation of guanosine tetraphosphate and guanosine pentaphosphate in Myxococcus xanthus during starvation and myxospore formation. J Bacteriol 141:297–304. doi:10.1128/jb.141.1.297-304.1980 6766441 PMC293584

[25] Harris BZ, Kaiser D, Singer M. 1998. The guanosine nucleotide (P)ppGpp initiates development and A-factor production in Myxococcus xanthus. Genes Dev 12:1022–1035. doi:10.1101/gad.12.7.1022 9531539 PMC316683

[26] Kaplan HB, Plamann L. 1996. A Myxococcus xanthus cell density-sensing system required for multicellular development. FEMS Microbiol Lett 139:89–95. doi:10.1111/j.1574-6968.1996.tb08185.x 8674994

[27] Jelsbak L, Søgaard-Andersen L. 2000. Pattern formation: fruiting body morphogenesis in Myxococcus xanthus. Curr Opin Microbiol 3:637–642. doi:10.1016/s1369-5274(00)00153-3 11121786

[28] Nariya H, Inouye M. 2008. MazF, an mRNA Interferase, mediates programmed cell death during multicellular Myxococcus development. Cell 132:55–66. doi:10.1016/j.cell.2007.11.044 18191220

[29] O’Connor KA, Zusman DR. 1991. Development in Myxococcus xanthus involves differentiation into two cell types, peripheral rods and spores. J Bacteriol 173:3318–3333. doi:10.1128/jb.173.11.3318-3333.1991 1904430 PMC207943

[30] Möller J, Luehmann T, Hall H, Vogel V. 2012. The race to the pole: how high-aspect ratio shape and heterogeneous environments limit phagocytosis of filamentous Escherichia coli bacteria by macrophages. Nano Lett 12:2901–2905. doi:10.1021/nl3004896 22591454

[31] Justice SS, Hunstad DA, Cegelski L, Hultgren SJ. 2008. Morphological plasticity as a bacterial survival strategy. Nat Rev Microbiol 6:162–168. doi:10.1038/nrmicro1820 18157153

[32] Chauhan A, Madiraju M, Fol M, Lofton H, Maloney E, Reynolds R, Rajagopalan M. 2006. Mycobacterium tuberculosis cells growing in macrophages are filamentous and deficient in FtsZ rings. J Bacteriol 188:1856–1865. doi:10.1128/JB.188.5.1856-1865.2006 16484196 PMC1426569

[33] Rosenberger CM, Gallo RL, Finlay BB. 2004. Interplay between antibacterial effectors: a macrophage antimicrobial peptide impairs intracellular Salmonella replication. Proc Natl Acad Sci U S A 101:2422–2427. doi:10.1073/pnas.0304455101 14983025 PMC356966

[34] Oliveira NM, Niehus R, Foster KR. 2014. Evolutionary limits to cooperation in microbial communities. Proc Natl Acad Sci U S A 111:17941–17946. doi:10.1073/pnas.1412673111 25453102 PMC4273359

[35] Sun X, Cai P, Sørensen SJ, Mortimer M, Gao C, Huang Q, Wang Y, Lin X, Wu Y, Zhu D, Chen R. 2020. Interspecific interactions in dual-species biofilms of soil bacteria: effects of fertilization practices. J Soils Sediments 20:1494–1501. doi:10.1007/s11368-019-02500-6

[36] Veening JW, Igoshin OA, Eijlander RT, Nijland R, Hamoen LW, Kuipers OP. 2008. Transient heterogeneity in extracellular protease production by Bacillus subtilis. Mol Syst Biol 4:184. doi:10.1038/msb.2008.18 18414485 PMC2387230

[37] Zusman DR. 1984. Cell-cell interactions and development in Myxococcus xanthus. Q Rev Biol 59:119–138. doi:10.1086/413780

[38] Veening JW, Hamoen LW, Kuipers OP. 2005. Phosphatases modulate the bistable sporulation gene expression pattern in Bacillus subtilis. Mol Microbiol 56:1481–1494. doi:10.1111/j.1365-2958.2005.04659.x 15916600

[39] Chung JD, Stephanopoulos G, Ireton K, Grossman AD. 1994. Gene expression in single cells of Bacillus subtilis: evidence that a threshold mechanism controls the initiation of sporulation. J Bacteriol 176:1977–1984. doi:10.1128/jb.176.7.1977-1984.1994 8144465 PMC205302

[40] Losick RM. 2020. Bacillus subtilis: a bacterium for all seasons. Curr Biol 30:R1146–R1150. doi:10.1016/j.cub.2020.06.083 33022258

[41] Veening JW, Stewart EJ, Berngruber TW, Taddei F, Kuipers OP, Hamoen LW. 2008. Bet-hedging and epigenetic inheritance in bacterial cell development. Proc Natl Acad Sci U S A 105:4393–4398. doi:10.1073/pnas.0700463105 18326026 PMC2393751

[42] Vlamakis H, Chai Y, Beauregard P, Losick R, Kolter R. 2013. Sticking together: building a biofilm the Bacillus subtilis way. Nat Rev Microbiol 11:157–168. doi:10.1038/nrmicro2960 23353768 PMC3936787

[43] Lord ND, Norman TM, Yuan R, Bakshi S, Losick R, Paulsson J. 2019. Stochastic antagonism between two proteins governs a bacterial cell fate switch. Science 366:116–120. doi:10.1126/science.aaw4506 31604312 PMC7526939

[44] Dannenberg S, Penning J, Simm A, Klumpp S. 2024. The motility-matrix production switch in Bacillus subtilis —a modeling perspective. J Bacteriol 206:e0004723. doi:10.1128/jb.00047-23 38088582 PMC10810213

[45] Norman TM, Lord ND, Paulsson J, Losick R. 2013. Memory and modularity in cell-fate decision making. Nature 503:481–486. doi:10.1038/nature12804 24256735 PMC4019345

[46] Dubnau D, Losick R. 2006. Bistability in bacteria. Mol Microbiol 61:564–572. doi:10.1111/j.1365-2958.2006.05249.x 16879639

[47] Smits WK, Kuipers OP, Veening JW. 2006. Phenotypic variation in bacteria: the role of feedback regulation. Nat Rev Microbiol 4:259–271. doi:10.1038/nrmicro1381 16541134

[48] Chastanet A, Vitkup D, Yuan GC, Norman TM, Liu JS, Losick RM. 2010. Broadly heterogeneous activation of the master regulator for sporulation in Bacillus subtilis. Proc Natl Acad Sci U S A 107:8486–8491. doi:10.1073/pnas.1002499107 20404177 PMC2889527

[49] Strauch MA, Wu JJ, Jonas RH, Hoch JA. 1993. A positive feedback loop controls transcription of the Spo0F gene, a component of the sporulation phosphorelay in Bacillus subtilis. Mol Microbiol 7:967–974. doi:10.1111/j.1365-2958.1993.tb01188.x 8483422

[50] Phillips ZEV, Strauch MA. 2002. Bacillus subtilis sporulation and stationary phase gene expression. Cell Mol Life Sci 59:392–402. doi:10.1007/s00018-002-8431-9 11964117 PMC11337539

[51] Gallegos-Monterrosa R, Kovács ÁT. 2023. Phenotypic plasticity: the role of a phosphatase family rap in the genetic regulation of Bacilli. Mol Microbiol 120:20–31. doi:10.1111/mmi.15060 37042030

[52] Fujita M, González-Pastor JE, Losick R. 2005. High- and low-threshold genes in the Spo0A regulon of Bacillus subtilis. J Bacteriol 187:1357–1368. doi:10.1128/JB.187.4.1357-1368.2005 15687200 PMC545642

[53] Barrows JM, Goley ED. 2023. Synchronized swarmers and sticky stalks: Caulobacter crescentus as a model for bacterial cell biology. J Bacteriol 205:e0038422. doi:10.1128/jb.00384-22 36715542 PMC9945503

[54] Newton A, Ohta N. 1990. Regulation of the cell division cycle and differentiation in bacteria. Annu Rev Microbiol 44:689–719. doi:10.1146/annurev.mi.44.100190.003353 2252398

[55] Paul R, Jaeger T, Abel S, Wiederkehr I, Folcher M, Biondi EG, Laub MT, Jenal U. 2008. Allosteric regulation of histidine kinases by their cognate response regulator determines cell fate. Cell 133:452–461. doi:10.1016/j.cell.2008.02.045 18455986 PMC2804905

[56] Del Medico L, Cerletti D, Schächle P, Christen M, Christen B. 2020. The type IV pilin PilA couples surface attachment and cell-cycle initiation in Caulobacter crescentus. Proc Natl Acad Sci U S A 117:9546–9553. doi:10.1073/pnas.1920143117 32295877 PMC7196804

[57] Chong TN, Panjalingam M, Saurabh S, Shapiro L. 2024. Phosphatase to kinase switch of a critical enzyme contributes to timing of cell differentiation. mBio 15:e0212523. doi:10.1128/mbio.02125-23 38055339 PMC10790692

[58] Paul R, Weiser S, Amiot NC, Chan C, Schirmer T, Giese B, Jenal U. 2004. Cell cycle-dependent dynamic localization of a bacterial response regulator with a novel di-guanylate cyclase output domain. Genes Dev 18:715–727. doi:10.1101/gad.289504 15075296 PMC387245

[59] Mann TH, Seth Childers W, Blair JA, Eckart MR, Shapiro L. 2016. A cell cycle kinase with tandem sensory PAS domains integrates cell fate cues. Nat Commun 7:11454. doi:10.1038/ncomms11454 27117914 PMC4853435

[60] Joshi KK, Bergé M, Radhakrishnan SK, Viollier PH, Chien P. 2015. An adaptor hierarchy regulates proteolysis during a bacterial cell cycle. Cell 163:419–431. doi:10.1016/j.cell.2015.09.030 26451486 PMC4600535

[61] Quon KC, Yang B, Domian IJ, Shapiro L, Marczynski GT. 1998. Negative control of bacterial DNA replication by a cell cycle regulatory protein that binds at the chromosome origin. Proc Natl Acad Sci U S A 95:120–125. doi:10.1073/pnas.95.1.120 9419339 PMC18146

[62] Kaczmarczyk A, Hempel AM, von Arx C, Böhm R, Dubey BN, Nesper J, Schirmer T, Hiller S, Jenal U. 2020. Precise timing of transcription by c-di-GMP coordinates cell cycle and morphogenesis in caulobacter. Nat Commun 11:816. doi:10.1038/s41467-020-14585-6 32041947 PMC7010744

[63] Tsokos CG, Perchuk BS, Laub MT. 2011. A dynamic complex of signaling proteins uses polar localization to regulate cell-fate asymmetry in Caulobacter crescentus. Dev Cell 20:329–341. doi:10.1016/j.devcel.2011.01.007 21397844 PMC3068846

[64] Childers WS, Xu Q, Mann TH, Mathews II, Blair JA, Deacon AM, Shapiro L. 2014. Cell fate regulation governed by a repurposed bacterial histidine kinase. PLoS Biol 12:e1001979. doi:10.1371/journal.pbio.1001979 25349992 PMC4211667

[65] Laub MT, Chen SL, Shapiro L, McAdams HH. 2002. Genes directly controlled by CtrA, a master regulator of the caulobacter cell cycle. Proc Natl Acad Sci U S A 99:4632–4637. doi:10.1073/pnas.062065699 11930012 PMC123699

[66] Tan W, Cheng S, Li Y, Li XY, Lu N, Sun J, Tang G, Yang Y, Cai K, Li X, Ou X, Gao X, Zhao GP, Childers WS, Zhao W. 2022. Phase separation modulates the assembly and dynamics of a polarity-related scaffold-signaling hub. Nat Commun 13:7181. doi:10.1038/s41467-022-35000-2 36418326 PMC9684454

[67] Lasker K, von Diezmann L, Zhou X, Ahrens DG, Mann TH, Moerner WE, Shapiro L. 2020. Selective sequestration of signalling proteins in a membraneless organelle reinforces the spatial regulation of asymmetry in Caulobacter crescentus. Nat Microbiol 5:418–429. doi:10.1038/s41564-019-0647-7 31959967 PMC7549192

[68] Saurabh S, Chong TN, Bayas C, Dahlberg PD, Cartwright HN, Moerner WE, Shapiro L. 2022. ATP-responsive biomolecular condensates tune bacterial kinase signaling. Sci Adv 8:eabm6570. doi:10.1126/sciadv.abm6570 35171683 PMC8849385

[69] Lu N, Duvall SW, Zhao G, Kowallis KA, Zhang C, Tan W, Sun J, Petitjean HN, Tomares DT, Zhao GP, Childers WS, Zhao W. 2023. Scaffold-scaffold interaction facilitates cell polarity development in Caulobacter crescentus. mBio 14:MBio doi:10.1128/mbio.03218-22 PMC10127582 36971555

[70] Skerker JM, Shapiro L. 2000. Identification and cell cycle control of a novel pilus system in Caulobacter crescentus. EMBO J 19:3223–3234. doi:10.1093/emboj/19.13.3223 10880436 PMC313932

[71] Schmidt JM. 1966. Observations on the adsorption of Caulobacter bacteriophages containing ribonucleic acid. J Gen Microbiol 45:347–353. doi:10.1099/00221287-45-2-347 5969754

[72] Ardissone S, Fumeaux C, Bergé M, Beaussart A, Théraulaz L, Radhakrishnan SK, Dufrêne YF, Viollier PH. 2014. Cell cycle constraints on capsulation and bacteriophage susceptibility. Elife 3:1–30. doi:10.7554/eLife.03587 PMC4241560 25421297

[73] Zhou X, Eckart MR, Shapiro L. 2021. A bacterial toxin perturbs intracellular amino acid balance to induce persistence. mBio 12:1–17. doi:10.1128/mBio.03020-20 PMC8545095 33622732

[74] Poindexter JS. 1964. Biological properties and classification of the Caulobacter group. Bacteriol Rev 28:231–295. doi:10.1128/br.28.3.231-295.1964 14220656 PMC441226

[75] Helm RR. 2018. Evolution and development of scyphozoan jellyfish. Biol Rev Camb Philos Soc 93:1228–1250. doi:10.1111/brv.12393 29446223

[76] Gasparini F, Manni L, Cima F, Zaniolo G, Burighel P, Caicci F, Franchi N, Schiavon F, Rigon F, Campagna D, Ballarin L. 2015. Sexual and asexual reproduction in the colonial ascidian Botryllus schlosseri. Genesis 53:105–120. doi:10.1002/dvg.22802 25044771

[77] Hallgren J, Jonas K. 2024. Nutritional control of bacterial DNA replication. Curr Opin Microbiol 77:102403. doi:10.1016/j.mib.2023.102403 38035509

[78] Stock AM, Robinson VL, Goudreau PN. 2000. Two-component signal transduction. Annu Rev Biochem 69:183–215. doi:10.1146/annurev.biochem.69.1.183 10966457

[79] Mascher T, Helmann JD, Unden G. 2006. Stimulus perception in bacterial signal-transducing histidine kinases. Microbiol Mol Biol Rev 70:910–938. doi:10.1128/MMBR.00020-06 17158704 PMC1698512

[80] Cashin P, Goldsack L, Hall D, O’Toole R. 2006. Contrasting signal transduction mechanisms in bacterial and eukaryotic gene transcription. FEMS Microbiol Lett 261:155–164. doi:10.1111/j.1574-6968.2006.00295.x 16907715

[81] Heppner DE, Eck MJ. 2021. A structural perspective on targeting the RTK/Ras/MAP kinase pathway in cancer. Protein Sci 30:1535–1553. doi:10.1002/pro.4125 34008902 PMC8284588

[82] Roberts PJ, Der CJ. 2007. Targeting the RAF-MEK-ERK mitogen-activated protein kinase cascade for the treatment of cancer. Oncogene 26:3291–3310. doi:10.1038/sj.onc.1210422 17496923

[83] Lemmon MA, Schlessinger J. 2010. Cell signaling by receptor tyrosine kinases. Cell 141:1117–1134. doi:10.1016/j.cell.2010.06.011 20602996 PMC2914105

[84] Rauch N, Rukhlenko OS, Kolch W, Kholodenko BN. 2016. MAPK kinase signalling dynamics regulate cell fate decisions and drug resistance. Curr Opin Struct Biol 41:151–158. doi:10.1016/j.sbi.2016.07.019 27521656

[85] Hinz AJ, Larson DE, Smith CS, Brun YV. 2003. The Caulobacter crescentus polar organelle development protein PodJ is differentially localized and is required for polar targeting of the PleC development regulator. Mol Microbiol 47:929–941. doi:10.1046/j.1365-2958.2003.03349.x 12581350

[86] Zhang C, Zhao W, Duvall SW, Kowallis KA, Childers WS. 2022. Regulation of the activity of the bacterial histidine kinase PleC by the scaffolding protein PodJ. J Biol Chem 298:101683. doi:10.1016/j.jbc.2022.101683 35124010 PMC8980812

[87] Zhou B, Schrader JM, Kalogeraki VS, Abeliuk E, Dinh CB, Pham JQ, Cui ZZ, Dill DL, McAdams HH, Shapiro L. 2015. The global regulatory architecture of transcription during the Caulobacter cell cycle. PLoS Genet 11:e1004831. doi:10.1371/journal.pgen.1004831 25569173 PMC4287350

[88] Lasker K, Mann TH, Shapiro L. 2016. An intracellular compass spatially coordinates cell cycle modules in Caulobacter crescentus. Curr Opin Microbiol 33:131–139. doi:10.1016/j.mib.2016.06.007 27517351 PMC5069156

[89] Chen JC, Hottes AK, McAdams HH, McGrath PT, Viollier PH, Shapiro L. 2006. Cytokinesis signals truncation of the PodJ polarity factor by a cell cycle-regulated protease. EMBO J 25:377–386. doi:10.1038/sj.emboj.7600935 16395329 PMC1383518

[90] Radhakrishnan SK, Thanbichler M, Viollier PH. 2008. The dynamic interplay between a cell fate determinant and a lysozyme homolog drives the asymmetric division cycle of Caulobacter crescentus. Genes Dev 22:212–225. doi:10.1101/gad.1601808 18198338 PMC2192755

[91] Perez AM, Mann TH, Lasker K, Ahrens DG, Eckart MR, Shapiro L. 2017. A localized complex of two protein oligomers controls the orientation of cell polarity. mBio 8:1–16. doi:10.1128/mBio.02238-16 PMC5347347 28246363

